# Corium lavas: structure and properties of molten UO_2_-ZrO_2_ under meltdown conditions

**DOI:** 10.1038/s41598-018-20817-z

**Published:** 2018-02-05

**Authors:** O. L. G. Alderman, C. J. Benmore, J. K. R. Weber, L. B. Skinner, A. J. Tamalonis, S. Sendelbach, A. Hebden, M. A. Williamson

**Affiliations:** 1 Materials Development, Inc, 3090 Daniels Court, Arlington Heights, IL 60004 USA; 2 X-ray Science Division, Argonne National Laboratory, Argonne, IL 60439 USA; 3 Mineral Physics Institute, Stony Brook University, Stony Brook, NY 11794-2100 USA; 4 Nuclear Engineering, Argonne National Laboratory, Argonne, IL 60439 USA

## Abstract

In the exceedingly rare event of nuclear reactor core meltdown, uranium dioxide fuel reacts with Zircaloy cladding to produce eutectic melts which can subsequently be oxidized by coolant/moderator water. Oxidized corium liquids in the *x*UO_2_·(100 − *x*)ZrO_2_ system were produced *via* laser melting of UO_2_-ZrO_2_ mixtures to temperatures in excess of 3000 K. Contamination was avoided by floating the droplets on a gas stream within an aerodynamic levitator and *in-situ* high-energy x-ray diffraction experiments allowed structural details to be elucidated. Molecular dynamics simulations well reproduced diffraction and density data, and show less compositional variation in thermal expansion and viscosity than suggested by existing measurements. As such, corium liquids maintain their highly penetrating nature irrespective of the amount of oxidized cladding dissolved in the molten fuel. Metal-oxygen coordination numbers vary with both composition and temperature. The former is due to mismatch in native values, *n*_UO_(*x* = 100) ≈ 7 and *n*_ZrO_(*x* = 0) ≈ 6, and the requirement for oxygen site stabilization. The latter provides a thermal expansion mechanism.

## Introduction

Multiscale modelling of material processes is essential for both forensic investigation of historically rare fission reactor core meltdowns, as well as development and design of future power reactors with improved safety and performance^1–3^. At the smallest, atomic length scales, molecular dynamics based on semi-empirical interatomic potentials remains a popular and powerful tool^4–8^, allowing access to wider length and time scales as compared to full quantum mechanical treatments of the electronic structure. Experimental validation of such simulations is of paramount importance^1^, but data at the extreme temperatures reached during core meltdown is scarce owing to the inherent practical challenges. Recently measurements pertaining to the atomistic structure of molten fuel and fuel-bearing phases have become possible^9–11^. Herein we seek to apply such techniques to the molten corium phase, obtained during extensive fuel-clad interaction, and to exploit these measurements by evaluating promising interatomic potentials which, in turn, are used to predict key structural and bulk physical properties including thermal expansion and viscosity. Melt viscosity in particular is an important property governing the spreading, and thereby the cooling, of corium in core-catchers^12^. The potentials and properties derived are suitable for larger scale simulations, including solid-melt interfaces between core materials, or to study the incorporation and behaviour of fission products. Moreover the predicted physical properties can be used to inform higher level finite element codes as part of multiscale reactor models.

**Figure 1:**
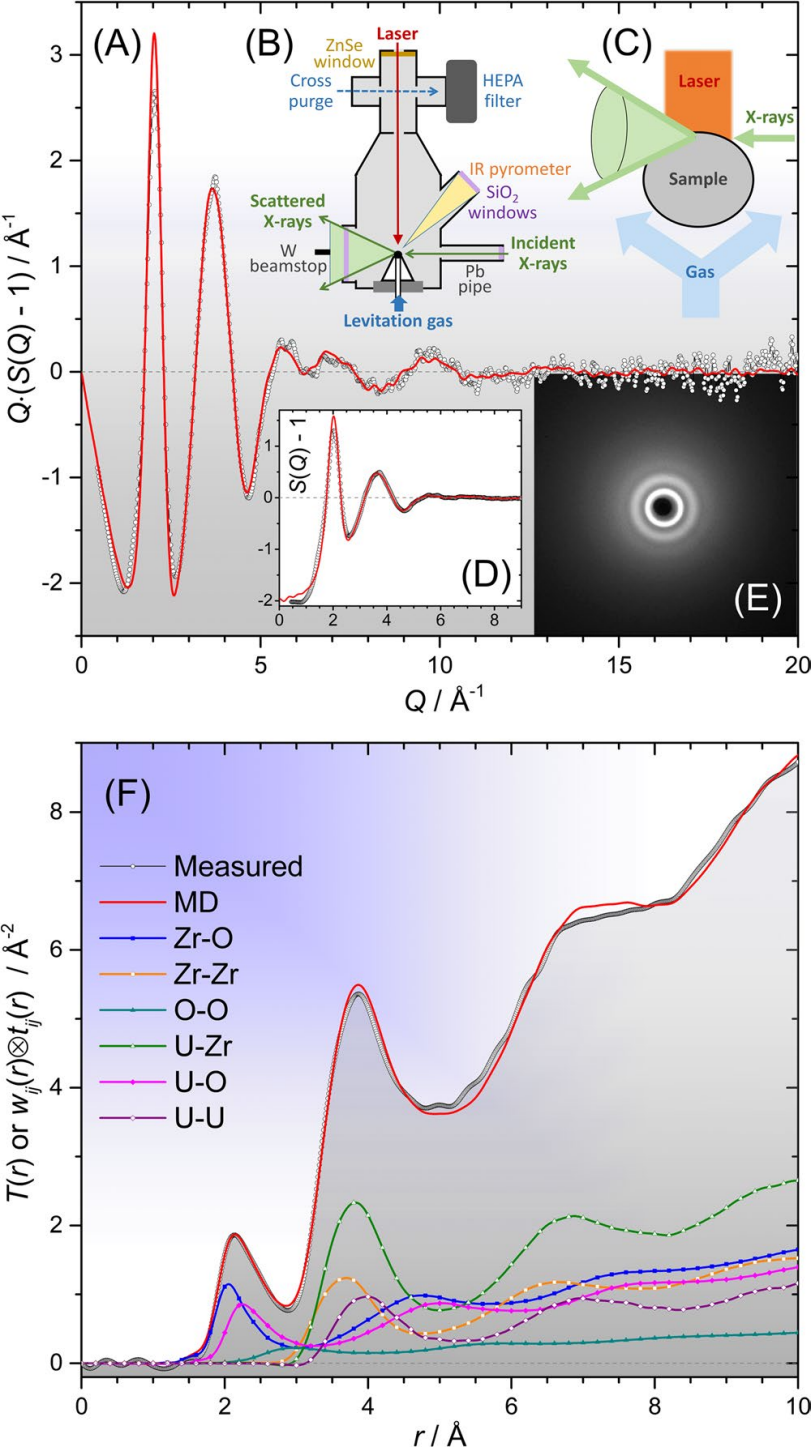
Comparison of measured (black circles) and MD modelled (red curves) x-ray diffraction data for liquid 27UO_2_·73ZrO_2_ at 3070 K. (**A**) Interference functions *Q*(S(*Q*) – 1), (**B**) schematic sample environment showing levitator within sealed chamber^10^, (**C**) close-up schematic of corium sample showing x-rays impinging on the top few hundred microns, (**D**) structure factors, S(*Q*) – 1, (**E**) close-up of two-dimensional data before integration of the above-plane scattering, (**F**) Fourier transforms of the data in (**A**) – the total correlation functions, *T*(*r*), as well as the weighted partial pair contributions *w*_*ij*_(*r*)⊗*t*_*ij*_(*r*). A *Q*_max_ = 17 Å^−1^ was used, without any *Q*-dependent window or modification function. The various reciprocal- and real-space functions are defined in^22,40^.

A severe accident scenario involving melting of the reactor core leads to the formation of corium lavas which are initially composed primarily of fuel and cladding materials. The most common such materials are based on UO_2_ and zirconium respectively. Subsequent interaction with water present as coolant and/or neutron moderator leads to oxidation of U-Zr-O liquids and H_2_ generation. In this paper, we study the structure and properties of oxidized molten corium in the UO_2_-ZrO_2_ pseudobinary obtained by laser heating ~100 mg quantities of ceramic material during levitation on a gas jet^13,14^ within a hermetically sealed chamber^9,10^, Fig. 1B. *In-situ* measurements using high-energy synchrotron x-rays yield the melt’s structure factors, *S*(*Q*), which provide atomistic level structural information used to verify the efficacy of molecular dynamics (MD) models, themselves based on interatomic potentials refined to similar measurements on the molten endmember dioxides^11,15^. The UO_2_ potentials employed herein are derived from those of Yakub, *et al*.^4^.

## Results and Discussion

**Figure 2:**
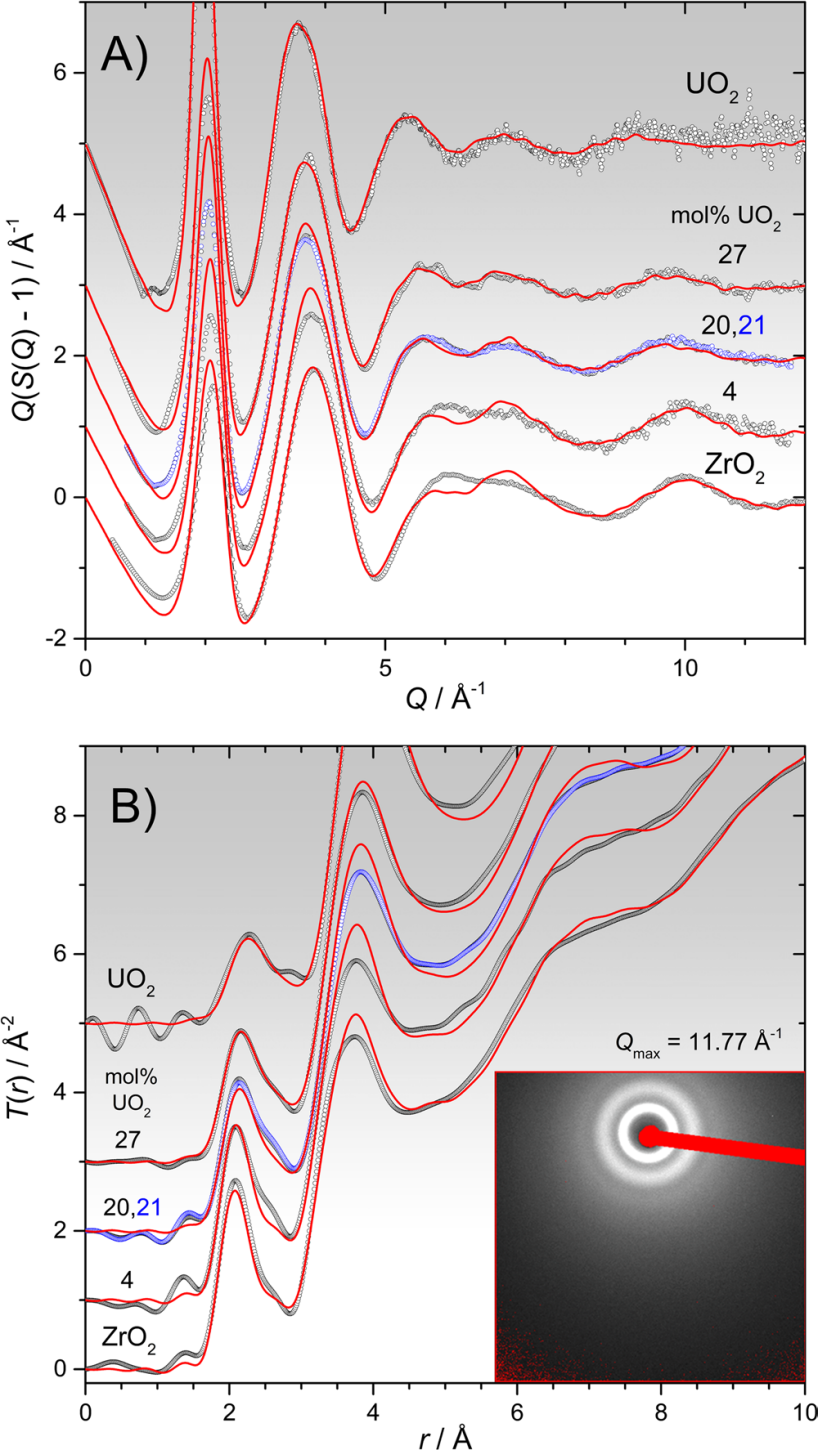
Stack plot comparisons of all six diffraction measurements (open points) and corresponding MD modelled functions (red curves), with measured compositions marked. (**A**) *Q*(S(*Q*) – 1), (**B**) *T*(*r*) with *Q*_max_ = 11.77 Å^−1^ and without any *Q*-dependent window or modification function. The inset shows the full detector panel with masked regions in red (mainly beamstop). In both parts A and B, the blue points correspond to the nominally 30 mol% UO_2_ sample measured in Ar:5%H_2_ which overlay those of the sample of the same nominal composition measured in Ar, demonstrating that the data are essentially indistinguishable.

The agreement obtained between modelled and experimental x-ray *S*(*Q*) and real-space Fourier transforms *T*(*r*) is excellent, Figs 1 and 2. The real-space goodness-of-fit parameters *Rχ*^16^ are reported in Table S1. *Rχ* is actually smallest for the binary 27 mol% UO_2_ melt, even compared to the pure endmembers (Figs S2 and S3), which may be attributed to the increased structural and chemical disordering arising from the presence of two different cationic species. The slightly larger *Rχ* for the 4, 20 and 21 mol% UO_2_ corium melts (Fig. 2) is likely due to a marginally higher mean UO_2_ content being represented in the x-ray measurements, as compared to the MD simulations. Since UO_2_ is volatilized throughout these short measurements, even integrating over only the last few seconds (Table S2) results in a time averaged UO_2_ content that is higher than that of the recovered material, at which MD simulations were run. This interpretation is supported by the excess intensity in the measured *T*(*r*) circa 2.55 Å due to U-O bonds (Fig. 2), and the fact that this increases in magnitude at earlier times. The data in Fig. 2 further indicate negligible differences between measurements made in Ar and highly reducing Ar:5%H_2_ gases (*cf*. 20 and 21 mol% UO_2_ samples). It is important to bear in mind that at such high temperatures, and at low oxygen potentials, the formation of low valence states of uranium, initially U^3+^, can be significant. However, previous work on UO_2-*x*_ melts^9^ revealed only subtle changes in the liquid diffraction patterns under different oxygen fugacities, from which a U^3+^ content of ≲ 27% was estimated in Ar:5%H_2_. Since U^4+^ is favored thermodynamically in Ar^9,17^ (owing to ppm levels of oxygen) and, based on the previous work, can be maintained kinetically even at lower oxygen potentials, we neglect the presence of lower valence states in our analyses.

**Figure 3:**
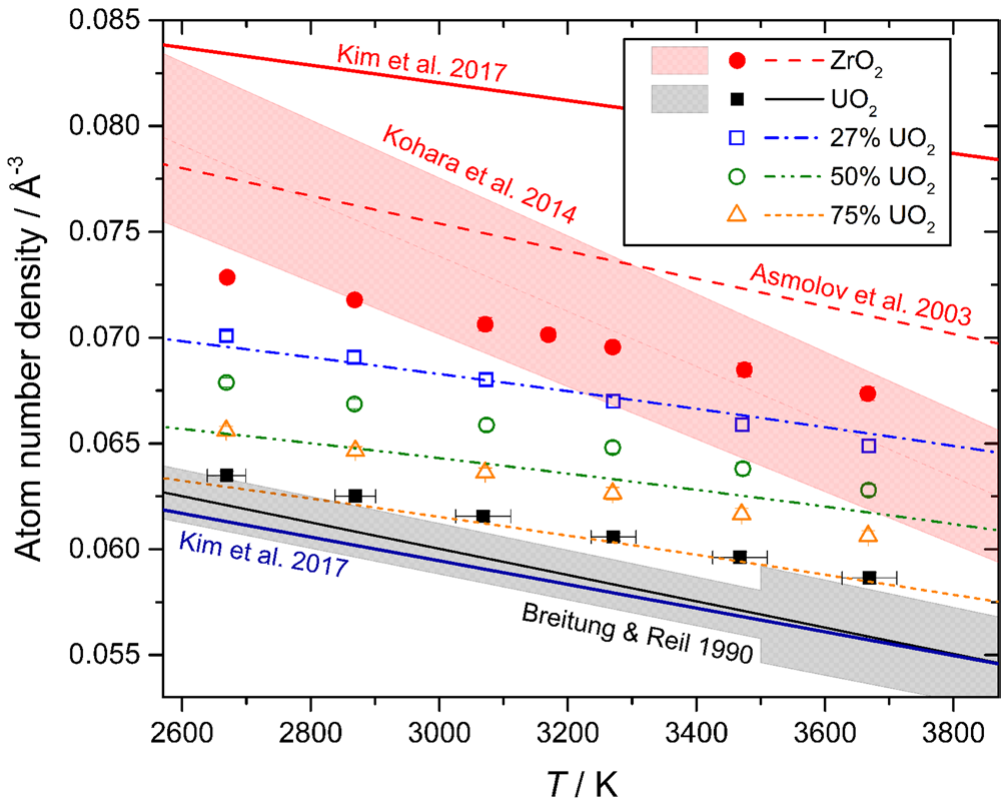
Densities of liquids in the *x*UO_2_·(100 − *x*)ZrO_2_ system. Symbols correspond to selected MD models of the present work. Molten UO_2_ density is that measured by Breitung and Reil^19^ (Pu bearing) in close agreement with Drotning^21^ and recommended by Fink^29^, with shaded region corresponding to the recommended uncertainty. Molten ZrO_2_ density is that measured by Kohara, *et al*.^18^ with uncertainty region shaded. The various broken curves correspond to the regular solution model of Asmolov, *et al*.^20^ which is constrained to be consistent with their recommendation for the density of molten ZrO_2_ (red dashed), the Breitung and Reil^19^ density, and their measurements for a 60.4 mol% UO_2_ corium liquid. The various literature relations have been extrapolated beyond the measured regions, including into the supercooled states, below the melting points. Temperature error bars on the UO_2_ MD model points are representative of those for the other MD models. Standard deviations in the modelled densities are similar to half the symbol heights. Recent MD results employing CRG EAM potentials from Kim, *et al*.^5^ are also shown. Note that we have plotted our own fits to the digitized data from Kim *et al*.’s Fig. 5 because their original Table 5 equations do not reproduce their graphical data and are erroneous.

Simulated melt densities were also obtained within reasonably good agreement with existing measurements^18–21^, Fig. 3, despite considerable uncertainty in the experimental density of molten ZrO_2_. The experimental verification of the MD models by x-ray diffraction and comparison of their densities to the literature, as well as their basis in the well-tested Yakub potentials for UO_2_^4,11^, encourages further interrogation of their statistical structure and properties.

### Composition Dependent Coordination Numbers

**Figure 4:**
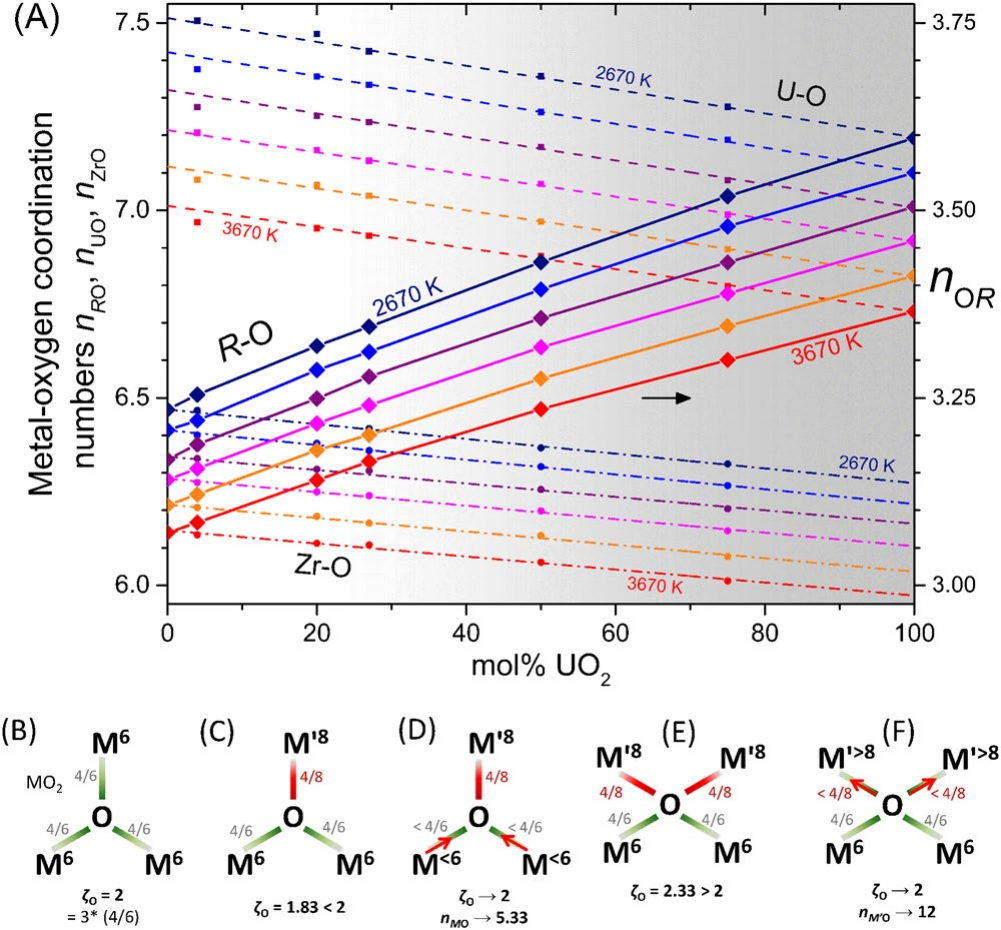
(**A**) Composition and temperature dependence of metal-oxygen coordination numbers in *x*UO_2_·(100 − *x*)ZrO_2_ liquids. Note that average *R*-O coordination is *n*_*R*O_ = *x*’*n*_UO_ + (1 − *x*’)*n*_ZrO_, with *x*’ = *x*/100 the mole fraction UO_2_. The right hand ordinate axis gives the oxygen-metal coordination *n*_O*R*_ = (1/2)*n*_*R*O_ and corresponds only to the average O-*R* coordination, and not to O-U or O-Zr which are given by *n*_O*j*_ = (*c*_*j*_/*c*_O_)*n*_*j*O_. Points correspond to the MD models, using radial cutoffs of 3.03 Å for Zr-O and 3.25 Å for U-O bonds. Broken curves are linear fits to the points (excluding *x* = 4 and 20 mol% UO_2_), solid curves connect the *n*_*R*O_ points which follow non-linear, quadratic trends owing to the fact *n*_UO_(*x*) and *n*_ZrO_(*x*) are not parallel. Six temperatures are represented, from 3670 K down to 2670 K in 200 K increments. **B-F**) Schema of oxygen site electrostatic bond strength sums (*ζ*_O_) in amorphous *M*O_2_ with native *n*_*M*O_ = 6, upon doping with *M’*O_2_, native *n*_*M’*O_ = 8. (**B**) Stable 3-fold oxygen site in an *M*O_2_ material with 6-fold cation coordination. (**C**) The 3-fold site becomes unstable when an 8-fold *M’* cation is substituted in. (**D**) Assuming the 8-fold cation is too large to reduce its coordination number to 6, in order to stabilize the 3-fold site some of the 6-fold cations reduce their coordination number. (**E**) As the *M’*O_2_ concentration increases, the average oxygen-cation coordination exceeds 3 and some 4-fold sites are necessary. These are also unstable without some adjustment of the cation environments. (**F**) To stabilize the 4-fold site, some cation coordination numbers must increase. These cannot be the *M* cations because they have decreased their (average) coordination to stabilize the 3-fold sites, and therefore the *M’*-O coordination must increase. See Fig. S7 for the complementary schema considering doping of *M’*O_2_ by *M*O_2_.

Several interesting phenomena are revealed in Fig. 4A, which shows that the cation-oxygen coordination numbers, *n*_ZrO_ and *n*_UO_, as well as their mean, *n*_*R*O_, depend on both composition and temperature (see also Fig. S4).

At high temperatures solid UO_2_-ZrO_2_ binaries form a cubic solid solution, with all cations coordinated to 8 oxygen ions. Further increasing the temperature leads to melting, upon which *n*_ZrO_ and *n*_UO_ have both been shown to *decrease* in the pure endmember dioxides^11,15,18^, as observed for several other oxides^15,22^, but importantly the effect is larger for zirconium. As such, molten ZrO_2_ has a different structure from molten UO_2_, akin to the difference in room temperature crystal structures, where monoclinic zirconia contains 7-fold Zr, while UO_2_ maintains the cubic structure with 8-fold U. The insight provided by our models of the binary melts is that this mismatch in endmember or ‘native’ *R*-O coordination numbers gives rise to linear composition dependence of both *n*_ZrO_(*x*) and *n*_UO_(*x*). Since the slopes ∂*n*_ZrO_/∂*x* ≠ ∂*n*_UO_/∂*x*, interpolation of *n*_*R*O_(*x*) between endmembers, Fig. 4A, is non-linear, and specifically, quadratic. A similar effect is observed for the alkali cations in chemically more complex mixed alkali silicate glasses^23,24^. Further support for the existence of this effect is provided by comparison of the appropriately weighted *g*_UO_(*r*) and *g*_ZrO_(*r*) taken from simulations of the endmember dioxides, to the x-ray diffraction data for the 27 mol% UO_2_ corium binary. Figures S5 and S6 show that use of the endmember cation-oxygen bond length distributions leads to poorer agreement with the experimental corium *T*(*r*) in the region of the first peak, than does the simulation of the binary itself (Fig. 1F). We propose that the variation in local structure with composition can be qualitatively understood in terms of the stabilization of the various oxygen environments present.

### Oxygen site stabilization

The concept of the Pauling electrostatic bond strength (EBS)^25^ has been shown to be helpful in understanding amorphous structures^26^. Within this approach the *M*-O bonds are assigned an EBS of *s* = *Z*_*M*_/*n*_*M*O_ and then the stability of the anionic oxygen sites are assessed by the proximity of the EBS sum,

1 $${\zeta }_{{\rm{O}}}={\sum }_{i=1}^{{n}_{{\rm{O}}M}}{s}_{i}\simeq |{Z}_{{\rm{O}}}|=2,$$

to its ideal value of |*Z*_O_| = 2, where the sum is taken over all O-*M* bonds to the given O^2−^ anion. Figure 4B shows a schematic representation of a 3-fold oxygen site in a material built up of only 6-fold *M*^4+^ cations, similar to molten zirconia. The oxygen must be on average 3-coordinated given the universal relationship *n*_*jk*_ = (*c*_*k*_/*c*_*j*_) *n*_*kj*_ where *c*_*k*_ and *c*_*j*_ are atomic fractions of the *k*^th^ and *j* ^th^ atomic species respectively, or *n*_O*M*_ = (1/2) *n*_*M*O_ for *M*O_2_ stoichiometry (Fig. 4A right hand ordinate). In this case, *ζ*_O_ = 2 and the site stability is maximized (Fig. 4B). Figure 4C to F illustrate the effect of introducing a larger cation, *M*’, with larger native *n*_*M*’O_(100) = 8. We have chosen the value 8, which is larger than *n*_UO_(100) ≈ 7, in order to simplify the discussion by means of an integer native O-*M*’ coordination of *n*_O*M*’_(100) = 4. Figure 4C considers the dilute case, where *M*’O_2_ is doped into the *M*O_2_ melt such that the most likely ‘mixed cation’ oxygen sites are 3-fold, with two *M* and a single *M*’. With native coordination numbers maintained, *ζ*_O_ = 1.83 < 2 and the site is less stable than in the undoped melt. One way to stabilize the site would be for the *M*’ cations to adopt the 6-fold coordination environments of the host *M* cations. However, this is not what we observe in the corium MD models, and if this mechanism is unavailable, then the only option for O site stabilization is for the *M* cations to reduce their coordination. This behavior is illustrated in Fig. 4D where the arrows illustrate the shortening of the *M*-O bonds, which typically accompany a reduction in coordination number. This is exactly what is observed in the lower left of Fig. 4A, where *n*_ZrO_(*x*) begins to decrease as uranium is introduced. As the *M*’O_2_ concentration increases, 4-fold oxygen sites become more abundant, Fig. 4E, and these can be stabilized *via* an *increase* in cation coordination. Since *n*_*M*O_(*x*) is already decreasing to stabilize the 3-fold oxygen sites, the only option available is for *n*_*M’*O_(*x*) to increase, as in Fig. 4F. This is the behavior observed in the upper left of Fig. 4A, where *n*_UO_(*x*) is larger than its native value at *x* = 100. One obtains a complementary picture if the dilution is reversed and *M*O_2_ is doped into the *M’*O_2_ melt, Fig. S7. The same general conclusions are reached in this way, whereby the right hand side of Fig. 4A is qualitatively described. Overall, for *∂n*_*R*O_/*∂x* > 0, the individual derivatives, *∂n*_*M*O_/*∂x* < 0 and *∂n*_*M’*O_/*∂x* < 0, must have the opposite sign.

Clearly the EBS approach is highly simplified, not all bonds to a given cation are equal, and in the actual melts there are wide bond-length distributions, Figs 1F and 2B, as well as distributions of coordination environments, Fig. S8. The flexibility introduced through variable bond lengths allows oxygen sites to be stabilized without necessarily any coordination changes. For example, a given cation can simultaneously form longer bonds to 4-fold oxygen sites, and shorter ones to 3-fold oxygen sites. The magnitude of the coordination changes within the EBS approach are suggested by the coordination numbers given in Figs 4D,F, S7C, S7E, which are those required to yield *ζ*_O_ = 2. We reason that bond length variability will significantly depress the magnitude of these coordination changes. Furthermore, if one iterates around Figs 4B–F or S7 there is positive feedback, i.e. the two cation coordination numbers simply diverge in a runaway process. The limiting mechanism preventing this is the existence of minimum and maximum stable coordination numbers, which are controlled by the form of the pair potentials. Finally, the effect is smaller in our MD simulations given the smaller difference in native coordination numbers (*n*_ZrO_(0) ≳ 6, *n*_UO_(100) ≈ 7) than illustrated. Nonetheless, similar non-ideal mixing behavior can be expected to occur in related (Ti,Zr,Hf)O_2_-(Th,U,Pu,…)O_2_ melts, but likely becomes negligibly small in more ideal cases, where cation radii are more closely matched, such as molten (U,Pu)O_2_ MOX fuels. In real Pu bearing melts, hypostoichiometry and the occurrence of reduced Pu^3+^ would play an important role, potentially again giving rise to composition dependent structures.

### Temperature Dependent Coordination Numbers and Thermal Expansion

**Figure 5:**
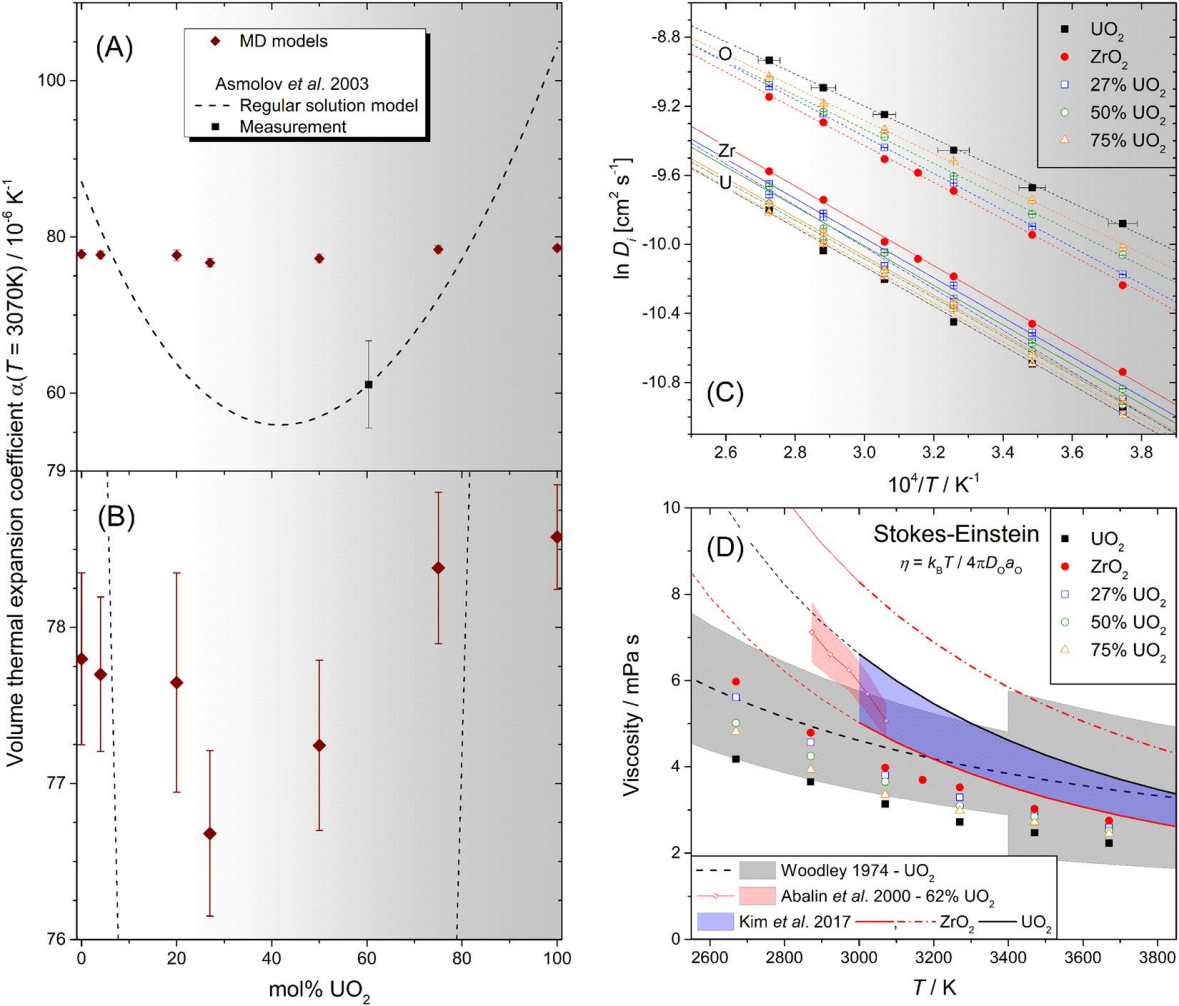
Physical properties of the MD modelled corium melts. (**A**,**B**) Volume thermal expansion coefficients at 3070 K compared to experimental data from Asmolov, *et al*.^20^ whose regular solution model is constrained to be consistent with the value for molten UO_2_ from Breitung and Reil^19^ (Pu bearing). (**A**) shows the same data as (**B**) over an extended ordinate range. (**C**) Self-diffusion coefficients plotted as ln *D*_*i*_ against 10^4^/*T* from which the activation energies, *E*_*i*_, and infinite temperature limits, *D*_0*i*_, can be obtained by linear regression of ln *D*_*i*_ = ln *D*_0*i*_ − *E*_*i*_/*k*_B_*T*. Temperature error bars on the UO_2_ MD model points are representative of those for the other MD models. The slopes yield the activation energies plotted in Fig. S9. (**D**) Viscosities estimated from the O^2−^ ion diffusivities using the Stokes-Einstein relationship, Eqn. 2, with slip boundary condition and hydrodynamic radius equal to the Shannon ionic radius *a*_O_ = 1.37 Å^44^. Similar estimates are obtained from the *M*^4+^ cation diffusivities as long as the hydrodynamic radius is set to encompass the first coordination shell^33–35^, Fig. S10. Statistical uncertainties are < 2%, within the size of the symbols. Measurements are also plotted; for molten UO_2_ from Woodley^30^ (extrapolated trend) with uncertainties recommended by Fink^29^ shaded, and for a 62 mol% UO_2_ corium melt from Abalin, *et al*.^31^. The latter authors report the kinematic viscosity, and we have therefore multiplied by the melt density (Asmolov, *et al*.^20^) to obtain the shear viscosities shown. The same viscosity data are plotted by Sudreau and Cognet^12^ with about 15% lower magnitude, presumably due to use of smaller densities, the provenance of which are not stated by those authors. Recent MD results from Kim *et al*^5^. are also shown, the shaded region corresponding to their range of interpolated corium viscosities (their Fig. 8c). The solid and chained curves for ZrO_2_ are based on Teter and EAM potentials, respectively. Note that we have plotted our own fits to the digitized data from Kim *et al*.’s Fig. 8a and b because their equations 38 and 39 (and their Table 5) are erroneous and do not reproduce their graphical data.

Also revealed in Fig. 4A is that *n*_ZrO_(*x*,*T*) and *n*_UO_(*x*,*T*) both decrease linearly with *T*, and this is plotted explicitly in Fig. S4, which reveals that the temperature coefficients are approximately independent of composition (Table S1). The values *∂n*_ZrO_/*∂T* ≈ −3.2(1)×10^−4^ K^−1^ and *∂n*_UO_/*∂T* ≈ −4.9(2)×10^−4^ K^−1^ are comparable to that calculated for titanium in molten TiO_2_, *∂n*_TiO_/*∂T* = −3.0(1)×10^−4^ K^−1^^22^, and it is evident that the magnitude of the effect increases with decreasing field strength, Ti^4+^ → Zr^4+^ → U^4+^, and increasing anharmonicity of the metal-oxygen pair potential. As with the thermal coordination number reduction, the temperature dependencies of the melt densities, Fig. 3, are also linear. This is suggestive of coordination change playing a key role as a thermal expansion mechanism. Positive correlation between coordination number and density is observed in many other settings, for example: the tetragonal and cubic phases of ZrO_2_ (8-fold Zr) are more dense than the monoclinic phase (7-fold Zr); both density and coordination numbers tend to increase with increasing pressure^27,28^; and materials like amorphous SiO_2_ and GeO_2_, composed of open networks of 4-fold tetrahedral cations, have lower atom number densities, despite their smaller cations, compared to melts of Ti, Zr, U dioxides, or the (U,Zr) corium melts, all of which contain more highly coordinated cations^11,15,18,22^. The calculated thermal expansion coefficients (TECs) of the corium melts at 3070 K are shown in Fig. 5A,B. They show remarkably little dependence on the corium composition, and lie between higher measured values for the endmembers^19,20^ and the lower value measured for a corium binary^20^. As such the models show no deep minimum in TEC as implied by the regular solution interpolation of Asmolov, *et al*.^20^. Such a deep minimum would require much larger structural variations with composition than observed by our diffraction measurements and MD simulations.

### Diffusivity and Viscosity

The self-diffusion coefficients calculated for the corium liquids are large, of order 10^−5^ to 10^−4^ cm^2^s^−1^ (Fig. 5C), similar to the Yakub, *et al*.^4^ model for UO_2_ upon which our potentials are based^11^. Since no measurements of *D*_*i*_ in corium liquids have been made, we have used the Stokes-Einstein (SE) relation to estimate the liquid viscosities from the *D*_O_:

2 $$\eta =\frac{{k}_{B}T}{4{\rm{\pi }}{D}_{{\rm{O}}}{a}_{{\rm{O}}}},$$

Figure 5D. The result for UO_2_ is slightly smaller than the recommended^29^ measurements made by Woodley^30^ in the 3140 to 3300 K range, although the agreement is reasonable given the experimental uncertainties and assumption of the SE relation. As with the TECs, little variation in viscosity is observed across the range of corium compositions, and little increase with ZrO_2_ content is observed, in contrast to that implied by the comparison of the corium measurement of Abalin, *et al*.^31^ to that of Woodley^30^. Abalin, *et al*. explain this discrepancy as due to build up of pressure within Woodley’s apparatus as *T* increases – leading to an apparent suppression of the activation energy for viscous flow. Note however that the difference between Abalin, *et al*.^31^ and Woodley^30^ (or our SE estimates) amounts to a factor ≲ 1.4 (1.8), which is small – smaller than the change in viscosity of liquid water between room temperature and its freezing point. Considering that liquid viscosity varies over at least 16 orders of magnitude, from 10^12^ Pas for supercooled liquids near their glass transition, down to 10^−4^ Pas for boiling or supercritical water, a factor of < 2 is of little practical importance. The less precise molten UO_2_ viscosity measurements of Tsai and Olander^32^ are in fact larger than Woodley’s, and similar to Abalin *et al*.’s corium measurement.

The key observation is that the corium liquids have incredibly low viscosities of only a few mPas, approaching that of cold water. Combined with their high mass densities, these fluids are highly penetrating and capable of traveling large distances within a reactor vessel during meltdown, independent of the amount of cladding incorporated into the molten fuel. This fluidity can enhance the spreading and cooling of melts within suitable core-catcher designs.

It is noteworthy that similar viscosities are obtained using the cationic self-diffusion coefficients in the Stokes-Einstein relation only if the diffusion of [*M*O_*n*_] coordination complexes are considered, such that the hydrodynamic radii are significantly larger than the ionic radii (Fig. S10). This has been explicitly validated in mixtures of molten halides^33–35^.

Arima^6^ has modelled other transport properties of corium melts, such as the thermal conductivities, and shown that they also show little variation with composition. Note however that the potential parameterizations used by Arima^6^ are based on those of Pedone, *et al*.^36^ (*ξ* = 0.60), which typically require further refinement to yield good agreement with liquid diffraction measurements at experimental temperatures^15,22^. Kim, *et al*.^5^ recently computed similar UO_2_ and ZrO_2_ melt viscosities to ours by means of the Green-Kubo stress autocorrelation approach (Fig. 5D). The authors used embedded atom method (EAM) many-body CRG potentials^7,8^, and additionally tested Teter pair potentials^37^ for ZrO_2_. They found some dependence on the choice of ZrO_2_ interatomic potential, but nonetheless inferred by compositional interpolation little variation in viscosity across the corium pseudobinary, in accord with our findings.

We note that EAM potentials, unlike simple pair potentials, allow for violation of the Cauchy relations between elastic tensor components, and have been shown^7^ to yield an improved bulk modulus for UO_2_ as compared to the Yakub model upon which our potentials are based. Direct comparisons of the EAM models^5^ to diffraction data for the molten end-members (Figs S11 to S13) reveal good agreement for UO_2_, but poorer agreement for ZrO_2_. Furthermore, whilst the density of molten UO_2_ appears well predicted by the EAM CRG potentials, that of ZrO_2_ is rather large, as compared to the measurements of Kohara, *et al*.^18^ and the function used by Asmolov, *et al*.^20^, see Fig. 3. The Teter potentials^37^ tested by Kim, *et al*.^5^ conversely yield a rather low melt density (very similar number density to UO_2_) and we have shown previously^22^ that these potentials require refinement to yield agreement with experimental densities and melt diffraction data.

## Conclusions

High energy x-ray diffraction from levitated droplets of molten corium has been used to validate classical molecular dynamics models of the same liquids. These models were based upon the well-tested UO_2_ interatomic potentials of Yakub *et al*.^4^ subsequently refined to similarly measured x-ray structure factors and densities of the UO_2_ and ZrO_2_ molten endmembers. Modelled properties including thermal expansion, self-diffusion coefficients and melt viscosities show very little dependence on U/Zr ratio in the UO_2_-ZrO_2_ pseudobinary, and confirm the extremely fluid and highly penetrating nature of these liquids. Corium liquids are therefore capable of traveling large distances within a reactor vessel during meltdown, independent of the amount of cladding incorporated into the molten fuel. Such high fluidity can be beneficial for spreading and cooling core melts within suitable core-catcher designs. Mismatch between the native metal-oxygen coordination numbers, *n*_UO_ ≈ 7 in molten UO_2_ and *n*_ZrO_ ≈ 6 in molten ZrO_2_, results in their variation with composition. In particular, both *n*_UO_ and *n*_ZrO_ decrease with increasing UO_2_ content, while their mean value increases. This behavior is explained on the basis of the stabilization of the various 3- and 4-fold oxygen sites present in the melts. Temperature dependent coordination numbers are also observed, and this is suggested to be a primary mechanism for liquid thermal expansion. Temperature derivatives ∂*n*_UO_/∂*T* ≈ −5 × 10^−4^ K^−1^ and ∂*n*_ZrO_/∂*T* ≈ −3 × 10^−4^ K^−1^, imply an increasing temperature dependence with increasing cation coordination and anharmonicity of the pair potentials. Our experimental verification of interatomic potentials for molten corium suggests their suitability for larger scale simulations, including melt-solid interface interaction, incorporation and behaviour of fission products, and for informing higher level finite element codes as part of multiscale reactor models.

## Methods

Diffraction experiments, using 100.23 keV x-rays, were performed at beamline 6-ID-D of the Advanced Photon Source, Argonne, Il, using an aerodynamic levitator, 400 W, 10.6 μm laser heating, and a hermetically sealed chamber, Fig. 1B,C, as described previously^9,10^. For a single sample formed *in-situ*, a sample to detector distance of 306.6 mm was used, whilst for later experiments on sintered ceramic beads, the distance was 837.7 mm, both of which were calibrated using a crystalline CeO_2_ standard. In the latter measurements a second ZnSe laser window was installed to increase redundancy in the containment of radioactive samples. The argon gas between the two windows was held at a slight overpressure such that a sensor could detect breach of either window and cut power to the laser.

One corium sample was produced by *in-situ* fusion of individual UO_2_ and ZrO_2_ ceramic beads in the levitator nozzle via laser melting. Based on the masses of the initial beads (28 mg ZrO_2_ and 69 mg UO_2_), a composition of 52.9 mol% UO_2_ was obtained, Table S2. However, due to the high vapor pressure of UO_2_ above the melt, significant enrichment in ZrO_2_ occurred during the *in-situ* synthesis of the bead. This compositional change was assumed to take place primarily during the initial stages of fusion, which was confirmed by comparison of the individual 1 s diffraction patterns collected during the final 27 s melting stage (Fig. S1). The diffraction pattern was observed to be invariant over the latter 16 s, and only these data were averaged together and analyzed. The composition of the recovered material was determined by inductively coupled plasma optical emission spectrometry (ICP-OES) to be 27(3) mol% UO_2_, and this composition was used for the analysis.

Further samples were made by milling together powders of −270 mesh (53 μm) depleted UO_2_ and 1 μm ZrO_2_. The mixtures were pressed into a tungsten die to form a pellet that was then sintered at 1700 ^◦^C for 1 hr in an argon atmosphere using an induction furnace. The pellets were removed from the die and ground using a 400 grit silicon carbide polishing wheel to form roughly spherical samples 2.5–3.0 mm in diameter, suitable for levitation. Details of three samples successfully measured in the molten state are recorded in Table S2. ICP-OES analyses reveal final sample compositions of 20, 21 and 4 mol% UO_2_, compared to their nominal values of 30, 30 and 15 mol% UO_2_ respectively, consistent with the high vapor pressure of UO_2_ and estimates based on the mass losses. Measurements on nominal compositions of 50 and 75 mol% UO_2_ were attempted but samples were only partially melted and are not reported here. The likely reasons for a lack of complete melting are the scattering of laser light by airborne particles of condensed volatilized UO_2_, along with the effective laser power loss associated with an additional laser window.

Either Ar or 5%H_2_:95%Ar levitation gases were used. Although the latter is highly reducing, the effects on diffraction patterns of molten UO_2−*x*_ have been shown to be small^9^, and are expected to be correspondingly smaller in dilutions with zirconia. This is confirmed by the strong similarity of the diffraction data for the two samples with the same nominal, 30 mol% UO_2_, compositions measured in different gases. As such, the UO_2_ component is considered stoichiometric in our analyses. Future investigations into the effect of U^3+^ content on melt structure and properties would be of great value, but extremely challenging experimentally, e.g. by *in-situ* U L_III_ edge XANES. Modelling of hypostoichometric melts on the other hand poses a more tractable avenue. The fully corrected structure factors, *S*(*Q*) – 1, are available as supplementary data online. Residual background intensity varying slowly with *Q* was removed using the top-hat convolution method (6.0 Å^−1^ top-hat width) and real-space intensity below 1.0 Å was supressed^38^, which is important for direct comparison to the MD simulated functions.

Atomistic models of corium melts were obtained by classical molecular dynamics (MD) simulation using the DL_POLY classic code^39^. Seven compositions were investigated, including the two endmembers, the ICP-OES measured final corium compositions of 4, 20 and 27 mol% UO_2_, and additionally 50 and 75 mol% UO_2_. As a starting point Morse potential parameterizations previously refined to measured x-ray structure factors of molten UO_2_ at 3270 K^11^ and molten ZrO_2_ at 3170 K^15^ were chosen. Those for UO_2_ are based on the well-tested Yakub potentials^4^, but with simplified interatomic pair-potentials taking the form

3 $${U}_{jk}(r)=\frac{{(\xi e)}^{2}{Z}_{j}{Z}_{k}}{4\pi {\varepsilon }_{0}r}+{E}_{jk}({(1-{e}^{-{a}_{jk}(r-{r}_{0jk})})}^{2}-1).$$

The second term is the short range Morse potential, parameterized by *E*_*jk*_, *a*_*jk*_ and *r*_0*jk*_, and the first term represents the Coulombic energy between ions of formal charge, *Z*_*j*_, in units of the electron charge, *e*, and 0 < *ξ* ≤ 1 is the ionicity parameter. Since the two starting models are incompatible due to their different *ξ* = 0.55 and 0.60, for UO_2_ and ZrO_2_ respectively, a new model was derived for liquid ZrO_2_ using *ξ* = 0.55 and identical O-O interactions to the UO_2_ model^11^. *E*_ZrO_ and *r*_0ZrO_ were refined to the x-ray *S*(*Q*) and measured density^18,20^, Fig. 3, within the isothermal-isobaric, *NPT* ensemble. The starting point for all MD trajectories was the 4800 atom configuration used in a study of molten TiO_2_^22^, which has the same stoichiometry, and similar atom number density (0.0726 Å^−3^) to the corium melts. The ZrO_2_ refinement runs began with 5 ps of equilibration (forces capped at 10^3^ *k*_B_*T*/Å), followed by a further 20 ps *NPT* trajectory, using a 1 fs timestep. A further 25 ps in the isothermal-isochoric *NVT* ensemble were used for calculation of the pair distribution functions, *g*_*jk*_(*r*)^40^, which were appropriately weighted, Fourier transformed and summed to yield the x-ray *S*(*Q*) (Figs S2, S3, 1,2). Ultimately the parameter *E*_ZrO_ was increased, resulting in an increase in system density toward the measured values^18,20^, Fig. 3, and *r*_0ZrO_ was adjusted to maintain the *U*_ZrO_(*r*) potential minimum at the same distance as that of the reference potential^15^. Any further increase in *E*_ZrO_, and thereby the density, had a detrimental effect on the agreement between measured and simulated x-ray *S*(*Q*), Fig. S3, which may explain the low density of 0.068 Å^−3^ obtained by Skinner, *et al*.^15^ during the original *ξ* = 0.60 refinement. The finalized potential parameters are given in Table S3. These were used for modelling of binary corium melts where U and Zr cations were decorated randomly onto the starting configuration^22^. For each composition the models were equilibrated at 3670 K for 50 ps followed by a further 50 ps (*NPT*) over which reported volume (density), mean-square-displacement (MSD) and coordination data were collected. The temperature was then reduced by 200 K and the procedure repeated (with just 25 ps equilibration) until *T* = 2670 K. Note that several of the lowest temperature points sampled correspond to supercooled liquids. This allows for interpolation of results through the melting points, as well as for isothermal comparisons across the full composition range. The liquidus temperature ranges from 3120 K (pure UO_2_), down to a minimum of 2850 K at 40 mol% UO_2_^41^. An additional 3170 K step was included for pure ZrO_2_ to match the x-ray experimental temperature^15^. A further 50 ps (*NVT*) was simulated for collection of *g*_*jk*_(*r*) for comparison to experimental *S*(*Q*), and to calculate coordination number and bond-angle distributions using the RINGS code^42^. In comparisons of x-ray and MD simulated *S*(*Q*) and *T*(*r*), the MD simulation closest to the experimental temperature (Table S2) was chosen, and the average density term, *T* ^0^(*r*) = 4π*ρ*_0_*r*, added to the experimental *T*(*r*) = *D*(*r*) + *T* ^0^(*r*), was based on the MD simulated atom number density, *ρ*_0_. Deviations from a statistical distribution of U and Zr cations were not detected, based on the observed U-U, Zr-Zr and Zr-U coordination numbers.

Note that any effects of charge transfer or disproportionation *via* 2U^4+^ ⇄U^3+^  + U^5+^ have been neglected in our simulations. Gillan^43^ estimates the free energy for this process in molten UO_2_ at Δ*F* = 0.7 to 1.7 eV which yields between 4% and 18% of each of the [U^3+^] = [U^5+^] populations at the melting point. Over the 1000 K *T* range of our simulations, these become 2–6% and 15–20%. Clearly this is non-negligible from the point of view of electronic processes, whereby small polaron hopping provides a conduction mechanism. However, we expect the effect on structure and bulk properties such as viscosity to be much weaker. Consider the success of our and previous approaches^4–7^ in correctly predicting a wide range of properties in the absence of charge transfer. Nonetheless future investigation into the effects of charge transfer is of interest. For example, models such as ours could be used as the basis for more accurate predictions of Δ*F* than those of Gillan^43^ who used the Ornstein–Zernike equation with hypernetted-chain closure relation to estimate the melt structure.

### Data availability

The fully corrected structure factors, *S*(*Q*) – 1, are available as supplementary data online. All other data are available upon request from the corresponding authors.

## Electronic supplementary material

Supplementary Information

Supplementary Dataset 1

Supplementary Dataset 2

Supplementary Dataset 3

Supplementary Dataset 4
