# Supplementary material for: Corium lavas: structure and properties of molten UO2-ZrO2 under meltdown conditions
Source: Sci Rep. 2018 Feb 5;8:2434. doi: 10.1038/s41598-018-20817-z (PMC5799198; doi:10.1038/s41598-018-20817-z)
Supplement: Supplementary file 1 — Supplementary Information [file 41598_2018_20817_MOESM1_ESM.docx]

**Supplementary Information**

**Corium lavas: structure and properties of molten UO_2_-ZrO_2_ under meltdown conditions**

O.L.G. Alderman,^1,2,a)^ C.J. Benmore,^2,b)^ J.K.R. Weber,^1,2^ L.B. Skinner,^2,3^ A.J. Tamalonis,^1^ S. Sendelbach,^1^ A. Hebden^4^ and M.A. Williamson^4^

^1^ *Materials Development, Inc., 3090 Daniels Court, Arlington Heights, IL 60004, USA*

^2^ *X-ray Science Division, Argonne National Laboratory, Argonne, IL 60439, USA*

^3^ *Mineral Physics Institute, Stony Brook University, Stony Brook, NY 11794-2100, USA*

^4^ *Nuclear Engineering, Argonne National Laboratory, Argonne, IL 60439, USA*

a) o.alderman@gmail.com

b) benmore@anl.gov

Figure S1: Pyrometry data for the corium sample formed by in-situ fusion of 28mg ZrO_2_ and 69mg UO_2_ beads in the levitator nozzle. The black curve is the raw apparent temperature, whilst the red curve (at higher *T*) is corrected for Fresnel losses and sample emissivity. The horizontal bars indicate times during which liquid x-ray diffraction patterns were collected and which part was later analyzed. The maximum sample temperature observed during fusion was 3431(20) K, whilst the mean *T* of the liquid over the data analysis window was 3070(20) K with a standard deviation of 31 K. The levitation gas used was 95%Ar:5%H_2_.

Figure S2: Comparison of measured (black circles ^1^) and MD modelled (red curves) x-ray diffraction data for liquid UO_2_ at 3270K. The interatomic potentials of Skinner, et al. ^1^, based on those of Yakub, et al. ^2^ were used. A) Interference functions *Q*(S(*Q*) – 1), with structure factors, S(*Q*) – 1, shown inset. B) Fourier transforms of the data in A) – the total correlation functions, *T*(*r*), as well as the weighted partial pair contributions *w_ij_*(*r*)⊗*t_ij_*(*r*). A *Q*_max_ = 17 Å ^-1^ was used, along with a Lorch modification function ^3^ to reduce the effects of high *Q* noise.

Figure S3: Comparison of measured (black circles ^4^) and MD modelled (red curves) x-ray diffraction data for liquid ZrO_2_ at 3170K. A) Interference functions *Q*(S(*Q*) – 1), with structure factors, S(*Q*) – 1, shown inset. B) Fourier transforms of the data in A) – the total correlation functions, *T*(*r*), as well as the weighted partial pair contributions *w_ij_*(*r*)⊗*t_ij_*(*r*). A *Q*_max_ = 17 Å ^-1^ was used, without any *Q*-dependent window or modification function.

Figure S4: Temperature and composition dependence of metal-oxygen coordination numbers in *x*UO_2_·(100 – *x*)ZrO_2_ liquids. Points correspond to the MD models, using radial cutoffs of 3.03 Å for Zr-O and 3.25 Å for U-O bonds. Solid lines are linear fits to the points.

Figure S5: Analogue of Fig. 1 in the main text, but using the *g*_UO_(*r*) and *g*_ZrO_(*r*) taken from simulations of the endmember dioxides at the same *T* = 3070 K. Comparison of measured (black circles) and MD modelled (red curves) x-ray diffraction data for liquid 27UO_2_·73ZrO_2_ at 3070K. A) Interference functions *Q*(S(*Q*) – 1), with structure factors, S(*Q*) – 1, shown inset. B) Fourier transforms of the data in A) – the total correlation functions, *T*(*r*), as well as the weighted partial pair contributions *w_ij_*(*r*)⊗*t_ij_*(*r*). A *Q*_max_ = 17 Å^-1^ was used, without any *Q*-dependent window or modification function. The various reciprocal- and real-space functions are defined in ^5,6^.

Figure S6: Running goodness-of-fit parameter *Rχ*(*r*_cut_), where *r*_cut_ is the upper bound interatomic distance used in calculation of *Rχ*, and the lower bound is 1 Å. The black curve is based on the comparison in Fig. 1B, whilst the red curve is based on Fig. S5B, using the *g*_UO_(*r*) and *g*_ZrO_(*r*) taken from simulations of the endmember dioxides at the same *T* = 3070 K. It can be seen that the latter gives a poorer fit (larger *Rχ*) than the former, in the first peak region, supporting the existence of composition dependent cation-oxygen environments, as observed in the simulations, Fig. 4A.


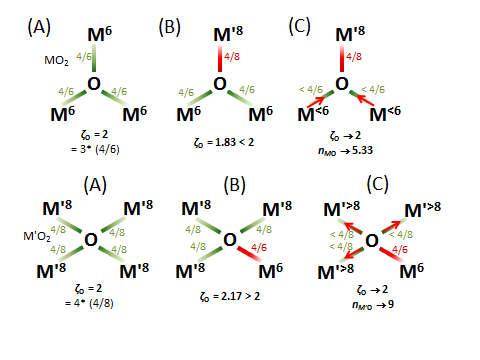

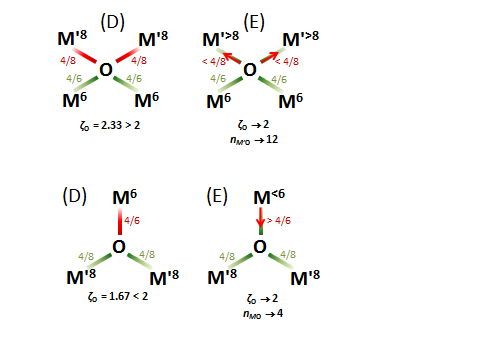


Figure S7: Schema of oxygen site electrostatic bond strength sums in amorphous *M’*O_2_ with native *n_M’_*_O_ = 8, upon doping with *M*O_2_, native *n_M_*_O_ = 6. (A) Stable 4-fold oxygen site in an *M’*O_2_ material with 8-fold cation coordination. (B) The 4-fold site becomes unstable when a 6-fold *M* cation is substituted in. (C) Assuming the 6-fold cation is too small to increase its coordination number to 8, in order to stabilize the 4-fold site some of the 8-fold cations increase their coordination number. (D) As the *M*O_2_ concentration increases, the average oxygen-cation coordination falls below 4 and some 3-fold sites are necessary. These are also unstable without some adjustment of the cation environments. (E) To stabilize the 3-fold site, some cation coordination numbers must decrease. These cannot be the *M’* cations because they have increased their (average) coordination to stabilize the 4-fold sites, and therefore the *M*-O coordination must decrease.

Figure S8: Oxygen site coordination number distribution in the MD model of molten 27UO_2_·73ZrO_2_ at 3270 K derived using the RINGS code ^7^. Radial cutoffs of 3.03 Å for Zr-O and 3.25 Å for U-O bonds were used. The most abundant sites are illustrated, the two most abundant 3-fold sites correspond to those in Fig. 4B to 4D of the main manuscript.

Figure S9: Activation energies for self diffusion, derived from the slopes of the fitted curves in Fig. 4C. Lines are to guide the eye.

Figure S10: Viscosities of molten UO_2_-ZrO_2_ from MD models estimated from the *M*^4+^ cation diffusivities using the Stokes-Einstein relationship for *M*O*_n_* complexes ^8-10^ with slip boundary condition and hydrodynamic radius equal to the 1^st^ minimum in *g_M_*_O_(*r*), 3.25 Å for U and 3.03 Å for Zr. Statistical uncertainties are < 2%, within the size of the symbols. Measurements are also plotted; for molten UO_2_ from Woodley ^11^ (extrapolated trend) with uncertainties recommended by Fink ^12^ shaded, and for a 62 mol% UO_2_ corium melt from Abalin, et al. ^13^. The latter authors report the kinematic viscosity, and we have therefore multiplied by the melt density (Asmolov, et al. ^14^) to obtain the shear viscosities shown. The same viscosity data are plotted by Sudreau and Cognet ^15^ with about 15% lower magnitude, presumably due to use of smaller densities, the provenance of which are not stated by those authors.

Figure S11: Comparison of measured (black circles ^1^) and MD modelled x-ray diffraction data for liquid UO_2_ at 3270K. The models are based on EAM CRG potentials (blue curves) from Kim, et al. ^16^ or interatomic potentials of Skinner, et al. ^1^, based on those of Yakub, et al. ^2^ (red curve). A) Interference functions *Q*(S(*Q*) – 1), with structure factors, S(*Q*) – 1, shown inset. B) Fourier transforms of the data in A) – the total correlation functions, *T*(*r*), as well as the weighted partial pair contributions *w_ij_*(*r*)⊗*t_ij_*(*r*). A *Q*_max_ = 17 Å ^-1^ was used, along with a Lorch modification function ^3^ to reduce the effects of high *Q* noise. Experimental data were transformed using the same density as the EAM CRG model.

Figure S12: Comparison of measured (black circles ^1^) and MD modelled x-ray diffraction data for liquid ZrO_2_ at 3170K. The models are based on EAM CRG potentials (blue curves) from Kim, et al. ^16^ or interatomic potentials of Skinner, et al. ^1^, based on those of Yakub, et al. ^2^ (red curve). A) Interference functions *Q*(S(*Q*) – 1), with structure factors, S(*Q*) – 1, shown inset. B) Fourier transforms of the data in A) – the total correlation functions, *T*(*r*), as well as the weighted partial pair contributions *w_ij_*(*r*)⊗*t_ij_*(*r*). A *Q*_max_ = 17 Å ^-1^ was used, along with a Lorch modification function ^3^ to reduce the effects of high *Q* noise. Experimental data were transformed using the same density as the EAM CRG model.

Figure S13: Running goodness-of-fit parameter *Rχ*(*r*_cut_), where *r*_cut_ is the upper bound interatomic distance used in calculation of *Rχ*, and the lower bound is 1 Å. The models and data used are those displayed in Fig. S2B, S3B, S11B and S12B.

Table S1: Results from MD models of corium melts. *Rχ* is a goodness-of-fit parameter ^17^ calculated from the differences between model and experimental *T*(*r*) over 1 ≤ *r* ≤ 8 Å.

| mol% UO_2_ | *Rχ* (%) | *T*_XRD_ (K) | XRD Ref. | *∂n*_ZrO_/*∂T*  (10^-4^ K^-1^) | *∂n*_UO_/*∂T*  (10^-4^ K^-1^) |
| --- | --- | --- | --- | --- | --- |
| 0 | 3.22 | 3170 | ^4^ | -3.28(7) | – |
| 4 | 3.88 | 2782 | This work | -3.29(4) | -5.21(19) |
| 20 | 3.68 | 2885 | This work | -3.19(6) | -5.07(10) |
| 27 | 2.57 | 3070 | This work | -3.14(8) | -4.92(6) |
| 50 | – | – | – | -3.04(9) | -4.81(4) |
| 75 | – | – | – | -3.12(5) | -4.80(5) |
| 100 | 4.40 | 3270 | ^1^ | – | -4.61(2) |

Table S2: Corium sample details. Compositions derived from the mass loss assume losses of the more volatile UO_2_ component only, and are based on the sample masses shown for before and after melting at the synchrotron beamline. Temperatures have their standard deviations shown in parentheses. Collection times indicate the time over which diffraction data indicate crystal (Bragg peak) free liquids and the time windows prior to quenching which were ultimately analyzed using the ICP-OES measured compositions (3^rd^ column).

| Composition / mol% UO_2_ | | | Mass / mg | | Gas | *T* / K  ± 20 | Collection time / s | | Density^14^  / gcm^-3^ |
| --- | --- | --- | --- | --- | --- | --- | --- | --- | --- |
| Nominal | Mass loss  ± 0.3 | Analyzed  ± 1 | Before | After |  |  | Liquid | Analyzed |  |
| 52.9 | n/a | 27 | 97 | n/a | Ar:5%H_2_ | 3070(31) | 27.0 | 16.0 | 6.13 |
| 30 | 21.3 | 21 | 99 | 81.3 | Ar:5%H_2_ | 2651(70) | 9.5 | 6.4 | 6.06 |
| 30 | 24.5 | 20 | 112 | 98.8 | Ar | 2885(74) | 5.7 | 2.7 | 5.94 |
| 15 | 4.2 | 4 | 88 | 69.5 | Ar | 2782(55) | 9.5 | 1.0 | 5.39 |

Table S3: Morse interaction parameters used for molecular dynamics simulations. The ionic charges, *ξZ_j_*, in units of the electron charge are given in the first column. U–O and O–O potentials are taken from ^1^, the Zr–O potential is adapted from ^4^ as described in the text.

| *j*-*k* ion pair | *E_jk_* / eV | *r*_0_*_jk_* / Å | *a_jk_* / Å^-1^ |
| --- | --- | --- | --- |
| U^2.2+^–O^1.1-^ | 0.21 | 2.880 | 1.50 |
| O^1.1-^–O^1.1-^ | 0.02 | 3.760 | 1.48 |
| Zr^2.2+^–O^1.1-^ | 0.30 | 2.493 | 1.80 |

**References**

1 Skinner, L. B. *et al.* Molten uranium dioxide structure and dynamics. *Science* **346**, 984-987, doi:10.1126/science.1259709 (2014).

2 Yakub, E., Ronchi, C. & Staicu, D. Molecular dynamics simulation of premelting and melting phase transitions in stoichiometric uranium dioxide. *J. Chem. Phys.* **127**, 094508 (2007).

3 Lorch, E. Neutron diffraction by germania, silica and radiation-damaged silica glasses. *J. Phys. C* **2**, 229 (1969).

4 Skinner, L. B. *et al.* Low cation coordination in oxide melts. *Phys. Rev. Lett.* **112**, 157801 (2014).

5 Keen, D. A. A comparison of various commonly used correlation functions for describing total scattering. *J. Appl. Cryst.* **34**, 172-177 (2001).

6 Alderman, O. L. G., Skinner, L. B., Benmore, C. J., Tamalonis, A. & Weber, J. K. R. Structure of Molten Titanium Dioxide. *Phys. Rev. B* **90**, 094204 (2014).

7 Le Roux, S. & Jund, P. Ring statistics analysis of topological networks: New approach and application to amorphous GeS_2_ and SiO_2_ systems. *Comp. Mater. Sci.* **49**, 70-83, doi:<http://dx.doi.org/10.1016/j.commatsci.2010.04.023> (2010).

8 Dewan, L. C., Simon, C., Madden, P. A., Hobbs, L. W. & Salanne, M. Molecular dynamics simulation of the thermodynamic and transport properties of the molten salt fast reactor fuel LiF–ThF_4_. *J. Nucl. Mater.* **434**, 322-327, doi:<http://dx.doi.org/10.1016/j.jnucmat.2012.12.006> (2013).

9 Salanne, M., Simon, C., Turq, P. & Madden, P. A. Conductivity− Viscosity− Structure: Unpicking the Relationship in an Ionic Liquid. *J. Phys. Chem. B* **111**, 4678-4684 (2007).

10 Brookes, R., Davies, A., Ketwaroo, G. & Madden, P. A. Diffusion coefficients in ionic liquids: relationship to the viscosity. *J. Phys. Chem. B* **109**, 6485-6490 (2005).

11 Woodley, R. E. The viscosity of molten uranium dioxide. *J. Nucl. Mater.* **50**, 103-106, doi:<http://dx.doi.org/10.1016/0022-3115(74)90066-X> (1974).

12 Fink, J. K. Thermophysical properties of uranium dioxide. *J. Nucl. Mater.* **279**, 1-18, doi:<http://dx.doi.org/10.1016/S0022-3115(99)00273-1> (2000).

13 Abalin, S. S. *et al.* Corium kinematic viscosity measurement. *Nucl. Eng. Des.* **200**, 107-115, doi:<http://dx.doi.org/10.1016/S0029-5493(00)00238-7> (2000).

14 Asmolov, V. G. *et al.* The Density of UO_2_–ZrO_2_ Alloys. *High Temp.* **41**, 627-632 (2003).

15 Sudreau, F. & Cognet, G. Corium viscosity modelling above liquidus temperature. *Nucl. Eng. Des.* **178**, 269-277, doi:<http://dx.doi.org/10.1016/S0029-5493(97)00137-4> (1997).

16 Kim, W. K., Shim, J. H. & Kaviany, M. Thermophysical properties of liquid UO_2_, ZrO_2_ and corium by molecular dynamics and predictive models. *J. Nucl. Mater.* **491**, 126-137, doi:<https://doi.org/10.1016/j.jnucmat.2017.04.030> (2017).

17 Grimley, D. I., Wright, A. C. & Sinclair, R. N. Neutron-Scattering from Vitreous Silica .4. Time-of-Flight Diffraction. *J. Non-Cryst. Solids* **119**, 49-64 (1990).
